# Reinke's edema

**DOI:** 10.4103/0971-3026.57212

**Published:** 2009-11

**Authors:** Sanjay Jain, Ravi Varma, Biswajyoti Hazarika, Sultan Pradhan, Asif Momin

**Affiliations:** Department of Radiology, Prince Aly Khan Hospital, Mumbai - 400 010, India

**Keywords:** Reinke's space, Computed Tomography

## Abstract

A unique imaging dilemma is presented where vocal fold thickening due to Reinke's edema mimicked tumor invasion of the vocal cords in a known case of thyroid malignancy.

## Introduction

Computed tomography of the neck is routinely performed in neck malignancies for disease staging. Soft tissue thickening of the vocal folds in the presence of an adjacent extralaryngeal malignancy is a worrying observation; however, it does not always signify laryngeal invasion, as is highlighted in the following case.

## Case Report

A 51-year-old man presented with a midline neck swelling and chronic hoarseness. On examination, a large prelaryngeal mass with a left level III lymphadenopathy was seen.

**Figure 1:**
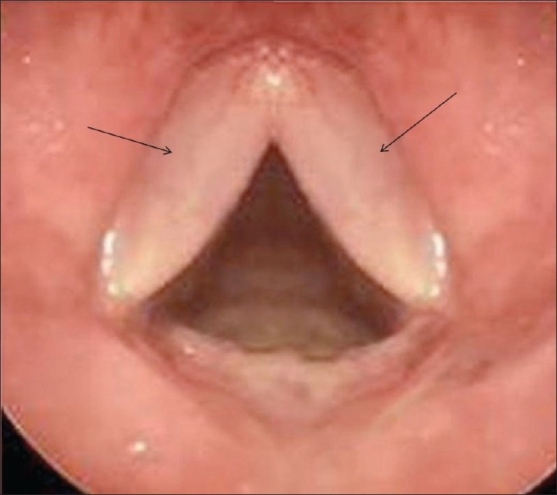
Endoscopic image showing edematous vocal cords (arrows)

**Figure 2:**
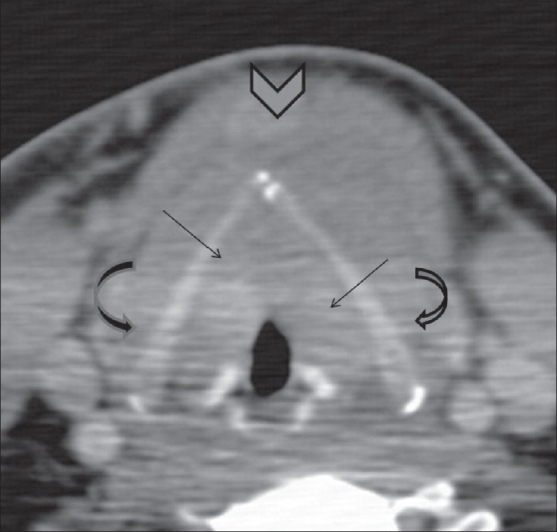
Axial CT scan at the level of the arytenoid cartilages shows diffuse soft tissue thickening of the vocal folds and paraglottic fat (arrows). The superior pole of the thyroid neoplasm is noted (arrowhead). Note that there is no evidence of thyroid cartilage invasion (curved arrows)

Laryngoscopy revealed edematous vocal cords with diminished vibrations [Figure 1]. Ultrasonography of the neck showed a midline, solid, heterogeneous mass arising from the thyroid isthmus with extrathyroidal extension into the strap muscles and thyroid cartilages and a large left level III metastatic lymph node. CT scan revealed diffuse infiltration of the paraglottic fat, which was suspicious of tumor infiltration [Figure 2]. However the thyroid and cricoid cartilages did not show any features of destruction. Biopsy of the mass revealed poorly differentiated small cell carcinoma of the thyroid.

At surgery, a large mass was seen arising from the isthmus and adjacent areas of both lobes, with extrathyroidal spread into the strap muscles and the anterior perichondrium of the thyroid cartilage; it was abutting the cricothyroid membrane. Multiple, enlarged, bilateral lymph nodes were seen. The patient underwent total thyroidectomy with excision of the strap muscles and bilateral modified neck dissection.

Histopathology of the resected specimen confirmed the preoperative diagnosis of poorly differentiated (insular) carcinoma of thyroid.

## Discussion

**Figure 3:**
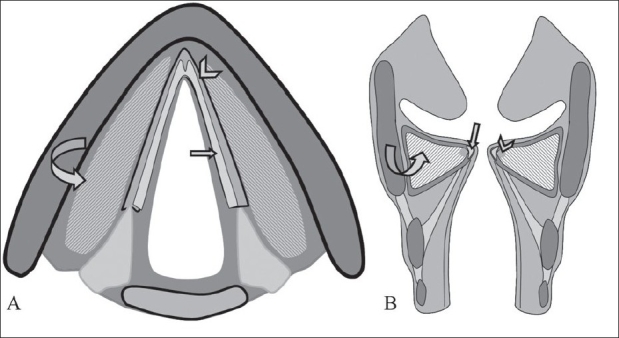
Diagram showing Reinke's space-Reinke's space (arrows), vocal ligament (arrowhead), vocalis muscle (curved arrow)

Reinke's space[1] is the subepithelial matrix of the vocal fold mucosa composed of elastin, collagen and other extracellular proteins; it is also known as the superficial lamina propria [Figure 3]. Reinke's edema is the accumulation of fluid in this space. The condition is fairly common and constitutes about 10% of benign laryngeal lesions.[2]

The edema develops by degrees, as a nonspecific reaction of the vocal folds to various irritative noxious agents. The main risk factor has been found to be cigarette smoking.[3] Other factors are chronic voice abuse, allergy, infection and laryngopharyngeal reflux; chronic sinusitis has also been implicated, though the association is not as noteworthy. Reinke's edema prevents normal vocal cord vibrations, giving rise to a hoarse, low-pitched voice.

Edematous swelling of the vocal cords can be easily recognized on indirect laryngoscopy, when the vocal cords are seen to be red and swollen and to have a slightly translucent appearance.[2] The reason this condition is important for radiologists, is that the presence of bilateral vocal cord edema on CT scan, may mimic cord involvement from adjacent malignancies, as in this present case, where the soft tissue raised the suspicion of tumor infiltration. This possibility in any case seemed unlikely in view of the preservation of the thyroid cartilage. However, the laryngoscopic findings were confirmatory and allowed us to confidently rule out cord involvement and to explain the cord thickening to be due to Reinke's edema.

To conclude, imaging is not required to diagnose Reinke's edema, as the diagnosis is evident on laryngoscopy. Nevertheless, it is important for the radiologist to be aware of this entity as the soft tissue thickening produced by Reinke's edema can be easily mistaken for tumor infiltration of the vocal cords.

## References

[1] Reinke FB (1895). Untersuchungen über das menschliche Stimmband. Fortschritte der Medizin, München,.

[2] van den Broek P, Hibbert J (1997). Acute and chronic laryngitis. Scott-Brown's Otolaryngology.

[3] Marcotullio D, Magliulo G, Pezone T (2002). Reinke's edema and risk factors: Clinical and histopathologic aspects. Am J Otolaryngol.

